# Characterization of cereal β-glucan extracts from oat and barley and quantification of proteinaceous matter

**DOI:** 10.1371/journal.pone.0172034

**Published:** 2017-02-14

**Authors:** Claudia Zielke, Ondrej Kosik, Marie-Louise Ainalem, Alison Lovegrove, Anna Stradner, Lars Nilsson

**Affiliations:** 1 Department of Food Technology, Engineering and Nutrition, Lund University, Lund, Sweden; 2 Department of Plant Biology and Crop Science, Rothamsted Research, West Common, Harpenden, United Kingdom; 3 European Spallation Source ESS AB, Lund, Sweden; 4 Department of Physical Chemistry, Lund University, Lund, Sweden; Henan Agricultural University, CHINA

## Abstract

An extraction method for mixed-linkage β-glucan from oat and barley was developed in order to minimize the effect of extraction on the β-glucan structure. β-Glucan were characterized in terms of molecular size and molar mass distributions using asymmetric flow field-flow fractionation (AF4) coupled to multiangle light scattering (MALS), differential refractive index (dRI) and fluorescence (FL) detection. The carbohydrate composition of the extracts was analysed using polysaccharide analysis by carbohydrate gel electrophoresis (PACE) and high-performance anion-exchange chromatography (HPAEC). Whether there were any proteinaceous moieties linked to β-glucan was also examined. Purified extracts contained 65% and 53% β-glucan for oats and barley, respectively. The main impurities were degradation products of starch. The extracts contained high molecular weight β-glucan (10^5^–10^8^ g/mol) and large sizes (root-mean-square radii from 20 to 140 nm). No proteins covalently bound to β-glucan were detected; therefore, any suggested functionality of proteins regarding the health benefits of β-glucan can be discounted.

## Introduction

The water soluble β-glucan from cereals, such as oat and barley, is a linear β-1,3 and β-1,4 linked polysaccharide formed solely of β-D-glucopyranosyl units. Several health effects of mixed-linkage β-glucan, acting as dietary fibre, have been approved by both, the U.S. Food and Drug Administration (FDA) [1,2] and the scientific panel of the EU (European Food Safety Authority (EFSA)) [3,4], including the reduction of serum cholesterol in the blood as well as reduced glycaemic and insulin responses [4]. Therefore, β-glucan is considered to be a functional and bioactive food ingredient which can reduce the risk of a number of chronic diseases with increased daily intake. Whilst these claims have been accepted, there is no understanding of the mechanism of action of β-glucan or potential ways to improve these health benefits in food for health purposes.

Some of the functional properties are believed to have their origin in the dissolution behaviour, particularly their capability of forming viscous solutions in the gut [5]. Self-association and formation of aggregates may additionally increase viscosity [6–9]. Previous observations showed a considerable variety in molar mass (M) and distribution of β-1,3 and β-1,4 linkages in β-glucan structure originating from different cereal origin [10]. Indeed, the high M and polydispersity of cereal β-glucan, indicating a presence of aggregates of the polysaccharides, makes their characterization challenging.

An interesting aspect with regard to the structural properties of β-glucan is the possible presence of β-glucan chains covalently bound to proteinaceous moieties as suggested previously [11,12]. These protein/ β-glucan links may play an important role as the presence of such moieties could influence the molecular interactions, solubility and aggregation behavior of the β-glucan. It has been reported that the addition of proteases to viscous oat flour slurries cause a decrease in viscosity indicating a relationship between protein content and viscosity [13]. Furthermore, Vårum & Smidsrød [14] found amino acid residues to be present in oat β-glucan preparations. Acker, Diemair & Samhammer [15] suggested that the presence of phosphate residues attached to oat β-glucan implied the presence of covalently bound proteins to the polysaccharide molecule. Therefore, the presence or absence of such moieties may explain the diversity of data on β-glucan functionality [11,16,17]. Furthermore, this could also influence M and viscosity of β-glucan preparations and might in turn lower low density lipoprotein (LDL) cholesterol values by forming viscous slurries in the gut [5]. These authors also showed, that high M β-glucan decreases LDL cholesterol in the blood more drastically then lower M β-glucan.

Although high proportions of β-glucan present in oat and barley is considered to be water soluble, there are still major problems with the extraction of β-glucan. Low yield, low purity and polysaccharide de-polymerization are common challenges. Under moderate conditions, e.g. water extraction at low temperatures, not all the β-glucan present is extracted, resulting in low yields. As discussed in detail by Webster & Woods [12], temperature, pH and choice of solvent may increase the yield of the β-glucan extraction. Similarly, increased yields can be obtained by using alkali solutions and increased temperature [18,19]. On the other hand, harsh extraction methods used for production of commercial production of mixed-linkage β-glucan can influence the structure and nature of the polysaccharide, for example, the ability to form aggregates [20], and such alterations in structure and properties may influence their possible health benefits. Therefore, it is of great interest to develop a methodology that would minimize the detrimental effects of extraction conditions on the β-glucan structure.

The main characterization method used in this study was asymmetric flow field-flow fractionation (AF4), a separation method especially well-suited for large and polydisperse polysaccharides owing to its broad separation range and gentle conditions. AF4 has, in recent years, been proven to be a valuable method for separation and characterization of food-related macromolecules [21] and colloidal structures [22]. The suitability of this method for the study of e.g. β-glucan and polysaccharides in beer [23] and cereal β-glucan macromolecules and aggregates has been described previously [6,24,25]. However, the methodology used in the present study had previously only been used for the study of cereal β-glucan standards [24,25]. The method described is considered to be gentle due to the relatively low shear forces to which the sample is exposed in the separation channel [22]. Separation is achieved by a longitudinal transport flow and a perpendicular field (cross-flow), which transports the analytes towards an accumulation wall in the AF4 separation channel [26]. As smaller particles have a higher rate of diffusion than larger ones, those will, at steady state, have a longer average distance from the accumulation wall. After this so-called relaxation step, elution commences and a longitudinal flow (showing a laminar flow profile due to the narrow channel height and relatively low flow rates) transports the analytes to the channel outlet. The smaller analytes with, on average, a longer distance from the accumulation wall will elute earlier due to the higher flow velocities experienced by analytes further from the accumulation wall. The larger analytes, being on average closer to the accumulation wall, will experience a lower flow velocity and will, thus, elute later. Hence, this results in size separation in what is referred to as Brownian mode separation.

The present study describes an extraction procedure for analysis of non-modified β-glucan from oat and barley. Mixed-linkage β-glucan was extracted from oat and barley flour of relatively high purity, considering the conditions of the method applied. The aim of the extraction method used, was to preserve the native properties of the β-glucan polymer such as possible aggregates or protein/ β-glucan links. No proteases were used during the extraction procedure in order to maintain any possible proteinaceous moieties. Polysaccharide analysis by carbohydrate gel electrophoresis (PACE) and high-performance anion-exchange chromatography (HPAEC) analysis of monosaccharide sugar composition were used to assess the purity of the β-glucan extracts. β-Glucan extracts were characterized using AF4 with multiangle light scattering (MALS) and differential refractive index (dRI) detection. Additional AF4 experiments were performed using in-line post-channel Calcofluor labelling for semi-quantitative fluorescence (FL) detection of β-glucan in the extracts. AF4-MALS-FL was also used for determination of the presence of proteinaceous moieties by pre-separation fluorescent labelling of peptide bonds.

## Materials and methods

### Substances and chemicals

Oat flour was from Oatwell CreaNutrition with a β-glucan content of 28% (w/w) (Swedish Oat Fiber AB, Väröbacka, Sweden). Barley flour was from Culinar ß-Fibre flour with a β-glucan content of 24% (w/w) (Culinar, Fjälkinge, Sweden). Both flours were rich in proteins and did not undergo any harsh treatment during production and therefore their structure was not substantially affected prior to these experiments. The acetone (technical grade) and ethanol (absolute grade) used during the extraction were purchased from VWR Chemicals, Fontenay sous Bois, France. The heat-stable α-amylase (from *Aspergillus oryzae*, ~30 U/ mg) was purchased from Sigma Aldrich, Darmstadt, Germany. For the preparation of the AF4 carrier liquid NaNO_3_ (Merck, Darmstadt, Germany) and NaN_3_ (BDH, Poole, UK) were used. Chemicals for fluorescence labelling were the following: Calcofluor fluorescent brightener 28 and EDAC (1-ethyl-3-(3-dimethylaminopropyl)carbodiimide) hydrochloride (Sigma Aldrich, Darmstadt, Germany), 7-methoxycoumarin-3-carboxylic acid (Invitrogen Molecular Probes, Thermo Fisher Scientific Inc., Waltham, Massachusetts, USA), tris(hydroxymethyl)-aminomethane (ICN Biomedicals Inc., Irvine, California, USA) and DMSO (Sigma Aldrich, Darmstadt, Germany). For enzymatic digestion prior to PACE, xylanase 11 or lichenase, were purchased from Prozomix Ltd., Haltwhistle, UK. The fluorophore ANTS (8-aminonaphtalene-1,3,6-trisulphonic acid) was purchased from Invitrogen Molecular Probes, Thermo Fisher Scientific Inc., Waltham, Massachusetts, USA. All other chemicals came from Sigma Aldrich, Darmstadt, Germany. Xylooligosaccharides (Xyl)_1–6_ were from Megazyme International Ltd., Bray, Co. Wicklow, Ireland. All experiments were performed using Milli-Q water (18.2 MΩ) from a Milli-Q system (Millipore Corp., Billerica, Massachusetts, USA).

### Extraction of β-glucan

In order to avoid proteases and harsh conditions, such as elevated pressure and alkaline treatments, the following procedure was used. The first extraction step involved defatting 2 g of flour with 75 mL acetone, stirring the suspension at RT for 2 h, followed by centrifugation for 20 min at 1,000 x *g*. The supernatant was discarded and the pellet dried overnight at RT. 1.5 g (~ 45 000 units) heat-stable α-amylase was re-suspended in 50 mL Milli-Q water and incubated at 100°C for 30 min to terminate any non α-amylase related exogenous activities [27]. The pre-treated α-amylase was mixed with the defatted flour in 100 mL Milli-Q water. The suspension was boiled for 1 h and stirred until the slurry had cooled to RT. 100 mL ethanol was added to precipitate the water soluble β-glucan and the sample stirred at RT for 1 h. After centrifugation for 30 min (2,000 x *g*), the precipitation step was repeated using the supernatant from the previous step. The pellets from both precipitations were re-dissolved in 50 mL of Milli-Q water, combined and heated to 65°C for 40 min. After cooling to RT, the viscous liquid was centrifuged for 1 h at 1,000 x *g* and the supernatant was oven-dried at 80°C for 15 h. The final extract was re-purified by repeating the whole procedure, from α-amylase digestion, a further two times. The amounts of α-amylase and solvents used were adjusted accordingly.

The β-glucan content of the extracts was analysed using the Mixed-Linkage β-Glucan Assay Kit from Megazyme International Ltd., Bray, Co. Wicklow, Ireland, with some modification in order to analyse the high concentration of β-glucan in the preparations produced. The first incubation step at 100°C was prolonged to 2 h. The amount of lichenase used in the next step was doubled, and the mixture incubated at 50°C for 2 h. The reaction solution was mixed with 100 mL of sodium acetate buffer (100 mM with a pH of 4) and stirred for 15 min before being centrifuged. The rest of the procedure was unchanged from that described in the kit. The developed procedure was verified by processing and measuring various samples with known high content of β-glucan. The final extracts used in this study contain 65% (w/w) β-glucan in case of oat and 53% (w/w) β-glucan in case of barley flour extract. The starch content of the extracts was analysed using the Total Starch Assay Procedure Kit, available as well from Megazyme.

### PACE (polysaccharide analysis by carbohydrate gel electrophoresis)

To check the quality of the extracted β-glucan and to identify possible impurities, PACE was performed as described elsewhere [28]. This method is used, among other applications, for elucidation of structure and characterization of carbohydrates. PACE relies on the migration of fluorophore-labelled mono- and oligosaccharides produced following digestion of a sample with specific glycosyl-hydrolases. The labelled sugars are separated based upon their size and structure. The fact that multiple samples can be run alongside each other on the polyacrylamide gels means that samples are exposed to exactly the same conditions during analysis.

Briefly, 250 μg aliquots of oat and barley extracts were prepared by drying stock solutions *in vacuo* (Savant Inc., Midland, Michigan, USA), they were then re-dissolved in water and digested with either xylanase 11 (4 μl ≈ 21.92 μg) or lichenase (1 μl ≈ 0.35 U) for 16 h at 40°C. The same amount of material was digested with α-amylase (2 μL ≈ 35.9 U) for 30 minutes at 50°C. The total reaction volume for all digestions was 500 μL. The digestion was terminated by boiling the sample for 30 min and samples dried *in vacuo*. Oat and barley fractions were labelled overnight with ANTS as described [28], as were xylo-oligosaccharides (Xyl)_1–6_ and α-amylase digested soluble starch. Following labelling, the derivatized sugars were dried *in vacuo* and reconstituted in 200 μL of 3 M urea before running on PACE gels. Digested soluble starch samples were diluted fourfold prior to loading on gel. PACE gels were visualized under UV light using a GelDoc-It TS2 imager (UVP, Jena, Germany) equipped with a GFP emission filter (513–557 nm).

### HPAEC-PAD analysis of monosaccharide sugar composition

HPAEC-PAD (high-performance anion-exchange chromatography equipped with pulse-amperometric detector) enables to study the monosaccharide composition of carbohydrates following acid hydrolysis. The monosaccharide analysis of β-glucan extracts was performed as described by Goubet et al. [29]. Briefly, 50 μg of either oat or barley β-glucan extract was incubated for 1 h at 121°C in 400 μl of 2 M TFA. The hydrolysates were lyophilised, re-suspended in water and analysed using a Dionex ICS3000 HPLC equipped with CarboPac PA 20 column (Thermo Fisher Scientific Inc., Waltham, Massachusetts, USA).

### AF4 analysis equipment and separation parameters

The AF4 instrument used was an Eclipse 3+ Separation System (Wyatt Technology Europe, Dernbach, Germany). The system was connected to a Dawn Heleos II multiangle light scattering (MALS) detector operating at 658 nm and an Optilab T-rEX differential refractive index (dRI) detector (both Wyatt Technology Europe) operating at the same wavelength. The carrier flow was delivered through an Agilent 1100 series isocratic pump with an in-line vacuum degasser and sample injection was via an Agilent 1100 series autosampler (Agilent Technologies, Waldbronn, Germany). To ensure that only particle free carrier liquid entered the system, a filter-holder with a 100 nm pore-size polyvinylidene fluoride (PVDF) membrane (Millipore Corp.) was placed between the pump and the channel inlet. The AF4 channel was a Wyatt long channel with a tip-to-tip length of 27.5 cm and a nominal thickness of 190 μm. The actual thickness was determined to be 156 μm by calibration with ferritin as described previously [30]. The ultra-filtration membrane forming the accumulation wall was made of hydrophilized polyethersulphone with a cut-off of 10 kDa (Microdyn-Nadir GmbH, Wiesbaden. Germany). 10 mM NaNO_3_ and 0.02% (w/v) NaN_3_ in Milli-Q water were used as carrier liquid. The samples were prepared at a concentration of 4 mg/ mL by dissolving the extracted β-glucan in the carrier liquid used for elution. The suspension was boiled for 30 min allowing the β-glucan to dissolve. The samples were cooled to RT and filtered through a 22 mm cellulose acetate membrane syringe filter with a 0.45 μm pore size (VWR International) prior to injection onto the channel. The sample was injected at 0.2 mL/ min for 4 minutes. The injected volume was 40 μl for oat and 80 μl for barley, corresponding to an injected mass of 160 μg and 320 μg, respectively. A focusing/ relaxation step of 5 min was performed prior to elution with a focusing flow rate of 0.5 mL/ min resulting in a void time (t^0^) of 9 min. The initial cross-flow rate during elution was 1 mL/ min followed by an exponential decay with a half-life of 4.5 min in order to avoid excessive retention and elution times. After elution, the AF4 channel was flushed without any cross-flow for 5 min to ensure a clean channel before analysis of the next sample. During the whole measurement the detector flow rate was kept constant at 0.5 mL/ min.

### Fluorescence measurements

The use of fluorescence detector enabled detection of both β-glucan and possible peptide residues through specific labelling of the purified β-glucan samples. A Jasco FP-1520 fluorescence detector (Jasco Inc., Easton, Maryland, USA) operating an Ushio Xenon Short Arc lamp (Ushio Inc., Tokyo, Japan) was used. Extracts were labelled in-line, post-channel with Calcofluor to detect β-glucan or pre-injection with EDAC to label peptide bonds, and analysed using the aforementioned AF4-MALS-FL set-up. For Calcofluor labelling of β-glucan, the method described by Ulmius, Önning, & Nilsson [25] was adopted, using a 25 mg/ L Calcofluor solution in 0.1 M tris(hydroxymethyl)-aminomethane buffer (pH 8), measuring the emission at λ_em_ = 445 nm (λ_ex_ = 415 nm). For labelling of possible proteinaceous matter in β-glucan extracts, the procedure described in [31] was adopted, with minor modification in that the labelling agent was diluted 1 in 10. Thus, 1 mM solution of EDAC in Milli-Q water was mixed for 3 hours with 1 mM solution of 7-methoxycoumarin-3-carboxylic acid in two parts of Milli-Q water and one part of DMSO to enhance the solubility. After mixing, the labelling solution was added to an already dissolved β-glucan sample in 1:1 ratio (v/v), stirred for 1 h and immediately injected onto the AF4 channel. The wavelengths used for detection of possible proteinaceous matter were λ_ex_ = 336 nm and λ_em_ = 402 nm.

### Elemental analysis

To measure the total protein content and other peptides that are possibly not associated with β-glucan, the extracts were analysed using Flash EA1112 elemental analyzer from Thermo Fisher Scientific, Delft, the Netherlands. The system uses the Flash Dynamic Combustion method, where complete combustion of the sample is reached through heating to 1000°C, and subsequently followed by determination of the produced elemental gases, in this case nitrogen from proteinaceous matter. Aspartic acid at three different concentrations was used to calibrate the instrument. The percentage of protein per sample (w/w) was estimated by multiplying the nitrogen content by a 6.25 nitrogen-to-protein conversion factor. The elemental analyzer has sensitivity down to 100 PPM.

### Data processing

Astra software in version 5.3.4.14 (Wyatt Technology Europe, Dernbach, Germany) was used to process the data obtained from the FL, MALS and dRI detectors following AF4 separation. M and root-mean-square radii (r_rms_) were calculated using the Berry method [32,33] performing a 1^st^ order fitting with the data obtained from the scattering detectors 9–14 (respective scattering angles: 69.3° - 121.2°). Lower and higher scattering angles were excluded as they did not correspond to the plot. The RI increment (d*n*/d*c*) used was 0.146 mL/ g as defined for β-glucan in aqueous solutions [34] whereas the second virial coefficient A_2_ was neglected.

## Results and discussion

The β-glucan extracts were analysed for chemical composition using a commercial kit (enzymatic β-glucan determination), PACE and HPAEC-PAD. AF4-MALS-dRI was used to obtain fundamental information regarding β-glucan structure such as M and r_rms_ distributions. Additional AF4-MALS-FL experiments were performed on the extracts to identify β-glucan, any possible polysaccharide-protein interactions and protein contaminations in the β-glucan fractograms.

An oat β-glucan extract of 65% (w/w) purity and barley β-glucan extract of 53% (w/w) purity were prepared. These obtained purities should be recognized as good bearing in mind the mild extraction conditions and methodology designed to retain the natural structure of the extracted polymers. Extraction methods using more extreme conditions, for example protease and alkali treatments, resulted in 57% (w/w) purity for oats [35], and up to 90% (w/w) purity [36] for barley extracts using acidic conditions (pH 4). Immerstrand et al. [35] also mentioned a potential influence of the geographical origin and growing conditions of the starting material on the gained extraction purities and yields when using the same extraction method. Extraction yields for similar conditions as in the present paper could not be found in literature.

The amount of starting material was 2 g of oat or barley flour of which 28% and 24% is known to be β-glucan. The theoretical maximum yield would therefore be 560 mg and 480 mg, respectively. Our yields were around 200 mg for oat with purity of 65% which leads to 130 mg of β-glucan, and 180 mg for barley with purity of 53%, leading to 95 mg of β-glucan. Therefore, in this study, the extraction efficiency is around 23% of total available β-glucan for oat and 20% for barley.

**Fig 1:**
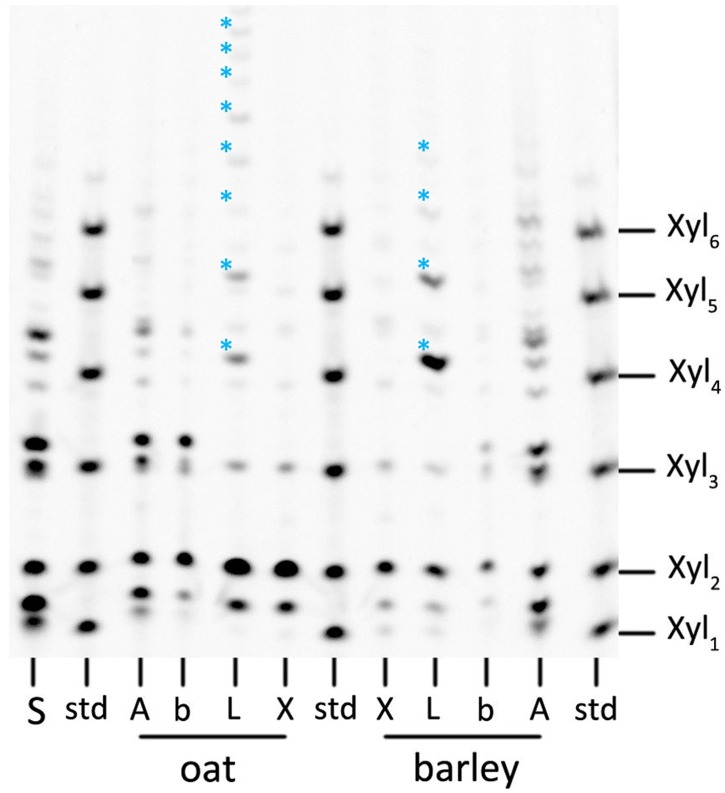
PACE gel. PACE gel showing extracted oat and barley mixed-linkage β-glucan, fingerprinted with specific glycosylhydrolases (‘A’–with α-amylase to analyse starch related impurities; ‘L’–with lichenase to analyse the presence and relative quantity of extracted β-glucan; ‘X’–with xylanase 11 to analyse xylan related impurities and ‘b’–background, non-digested sample) and their separation based on size and structure after derivatization with ANTS. ‘S’ shows starch sample digested with α-amylase and ‘std’ shows (Xyl)_1-6_ standards used for oligosaccharides identification. Bands marked with blue asterisks are mixed-linkage β-glucan specific oligosaccharides DP ≥ 3. Xyl_1_ –xylose, Xyl_2_ –xylobiose, Xyl_3_ –xylotriose, Xyl_4_ –xylotetraose, Xyl_5_—Xylopentaose, Xyl_6_ –Xylohexaose. Shorter oligosaccharides migrate further in the polyacrylamide gel.

The PACE gels of enzymatically digested oat and barley extracts with specific glycosyl-hydrolases are shown in Fig 1. Xylose to xylo-hexose standards (Xyl)_1-6_ (labelled as ‘std’) and α-amylase digested soluble starch (labelled as ‘S’) were run alongside other digests to identify the main impurities present in oat and barley β-glucan extracts. ‘b’ are blanks; these samples were incubated with no addition of glycosyl-hydrolases. Even without digestion steps (lanes labelled ‘b’) small oligosaccharides are present, some of which co-incide with oligosaccharides released by amylase (A), indicating some remaining starch in the oat and barley preparations. ‘X’ indicates a sample digested with xylanase 11 showing (arabino)xylan-derived oligosaccharides and ‘L’ refers to samples digested with lichenase showing β-(1,3)(1,4)-glucan related oligosaccharides. The position and the intensity of the labelled sugars/ bands are similar for both oat and barley extracts, although there is clearly more G3 oligosaccharide present in barley than in oats and there are higher DP glucan oligosaccharides present in the oat digest. Mixed-linkage glucan oligosaccharides are marked with blue asterisks in Fig 1. ‘A’ is the sample digested with α-amylase, showing starch related impurities in the oat and barley extracts.

To obtain additional information of the extracts’ purity, HPAEC-PAD analysis of monosaccharide sugar composition was performed. The results are summarized in Table 1. On a dry weight basis, 92.16% (w/w) of the hydrolyzed oat extract is glucose while for the hydrolyzed barley extract the glucose content is 72.07% (w/w). In order to compare these values with the β-glucan content obtained using the commercially available β-glucan assay kit, from which values of 65% and 53% (w/w) for oat and barley, respectively, were obtained, the values need to be recalculated to correct for the difference in M (i.e. glucose: 180 g/ mol and glucose pyranosyl unit in a β-glucan chain: 162 g/ mol). Thus, a correction factor (162 [g/ mol]/180 [g/ mol]) needs to be multiplied with the glucose content in order to estimate how much of that glucose originates from β-glucan. The results show that approximately 17% (oat) and 12% (barley) of glucose does not come from β-glucan. The starch content of the samples was determined to be 11% (w/w) each in both extracts using a commercial starch assay kit (Megazyme).

**Table 1:** Summary of characterization of the β-glucan extracts from oat and barley. The table includes purity and composition values, weight-average molar mass, average r_rms_ and mass recovery from the AF4 channel.

β-glucan source	β-glucan content [%(w/w)]	Starch content [%(w/w)]	content in extracts [%(w/w) of dry mass]						M_w_ [g/mol]^a^	r_rms_ [nm]^b^	Mass recovery [%]^c^
			glucose	xylose	arabinose	mannose	fucose	galactose			
oat	65	11	92.16	0.72	0.44	0.22	0.40	0.12	5.6·10^6^	96	72
barley	53	11	72.07	1.49	0.40	0.69	---	0.06	2.4·10^6^	82	98

^a^ Weight-average molar mass

^b^ z-average

^c^ from the FFF channel, recovery of total injected mass, measured mass (integrated dRI-signal representing the concentration over the fractogram) / injected mass

The origin of the remaining glucose is most likely related to various dextrins originating from degradation of starch during the extraction. Hydrolysis of starch by α-amylase can result in various oligosaccharides and dextrins of which the composition depends on the substrate and the specific α-amylase utilized [37]. The solubility of these enzymatic products is relatively low in ethanol [38,39] and they may precipitate with the β-glucan during the extraction. The results from PACE analysis also indicate this. Other non-glucose monosaccharides were found in very small quantities in the hydrolyzed oat and barley extracts (e.g. xylose, arabinose, mannose and galactose). Rhamnose and galacturonic acid were not detected in any sample. Hence, the main impurities of the extracted samples, not being starch, are other water soluble polysaccharides or sugar residues, coming from other cereal polysaccharides and hemicelluloses e. g. glucomannans, arabinoxylans and starch dextrins in the endosperm or the cell walls of the oat and barley grains.

**Fig 2:**
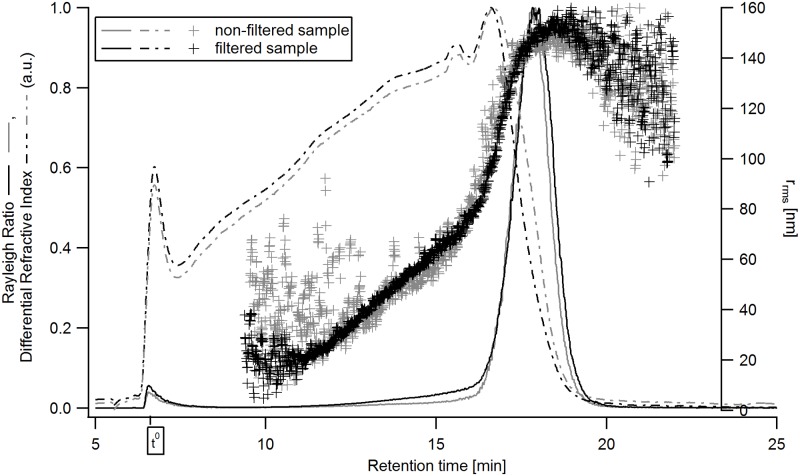
Fractogram of the β-glucan extract from oat, non-filtered and filtered sample. The signals are: grey solid line—Rayleigh ratio from MALS (a.u., 69.3° - 121.2°) from non-filtered sample, black solid line—Rayleigh ratio from MALS (a.u., 69.3° - 121.2°) from filtered sample, grey dashed line—dRI signal (a.u.) from non-filtered sample, black dashed line—dRI signal (a.u.) from filtered sample, grey crosses—r_rms_ of non-filtered sample, black crosses—r_rms_ of filtered sample. Channel and flow conditions as described in Materials and Methods, part e, except: initial cross-flow of 0.5 mL/ min with an exponential decay with half-life of 2 min, t^0^ = 6.5 min.

For further characterization of the β-glucan structure, AF4-MALS-dRI was utilized. Prior to the injection of the sample onto the AF4-channel, the prepared samples were filtered through a 0.45 μm pore size syringe filter with a cellulose acetate membrane. Sample filtering did not have any significant influence on the results but improved the signal-to-noise ratio as shown in Fig 2. These measurements were performed as pre-experiments and the flow conditions were different, resulting in different elution times compared to subsequent experiments reported in this study. The fractogram shows Rayleigh ratio signal (intensity of the scattered light) from the MALS detector and the dRI signal representing the concentration over the elution time as well as the calculated distribution for r_rms_. The MALS and dRI signals were normalized against the peak maxima. The results (Fig 2) show that the signals and size distributions remain similar with a mass recovery from the AF4 channel of 99% for the non-filtered and 96% for the filtered sample.

It is also evident that the noise in the determined r_rms_ distribution is considerably lower in the filtered sample, especially for sizes below 60 nm. The higher noise in the non-filtered sample is most likely originating from very low amounts of very large analytes which co-elute in the steric/ hyperlayer mode of AF4 [40]. The presence of even very low amounts of large species may give rise to noisy MALS signals impairing the size determination [41,42].

**Fig 3:**
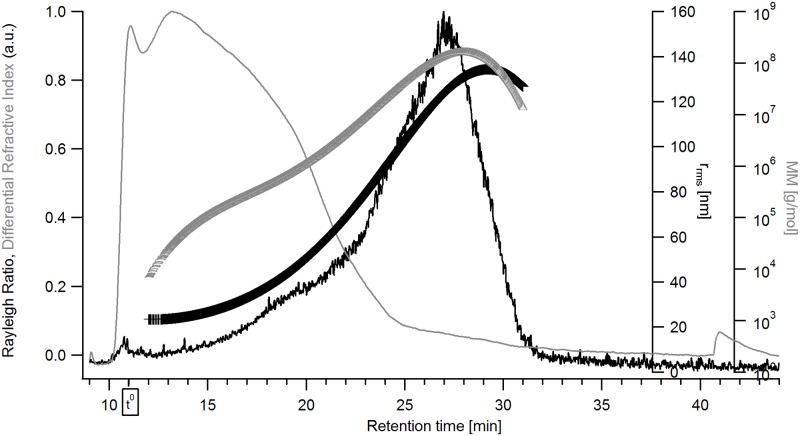
Fractogram of the β-glucan extract from oat. The signals are: black line—Rayleigh ratio from MALS (a.u., 69.3° - 121.2°), grey line—dRI signal (a.u.), grey triangles—molar mass distribution [g/mol], black crosses—r_rms_ distribution [nm].

**Fig 4:**
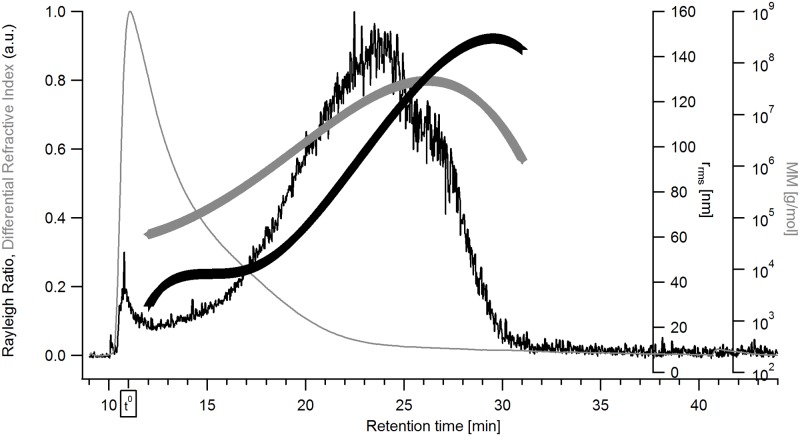
Fractogram of the β-glucan extract from barley. The signals are: black line—Rayleigh ratio from MALS (a.u., 69.3° - 121.2°), grey line—dRI signal (a.u.), grey triangles—molar mass distribution [g/mol], black crosses—r_rms_ distribution [nm].

**Fig 5:**
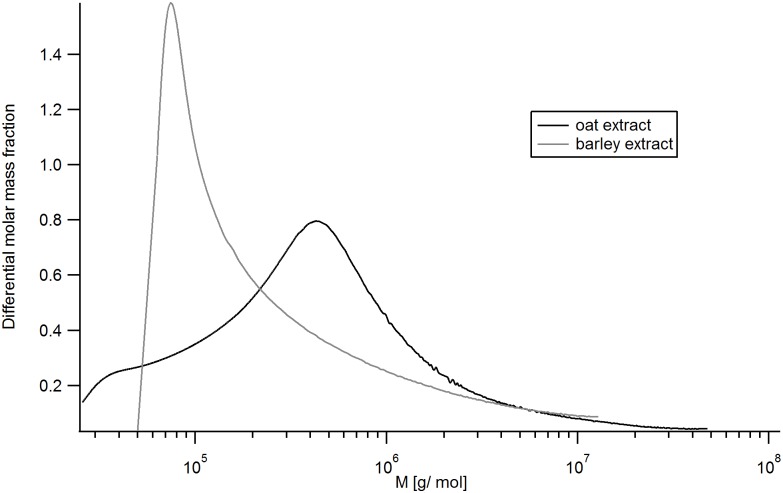
Differential molar mass fraction of the extracts. The colors denote: black—oat, grey—barley.

The plots in Figs 3 and 4 display fractionation of the extracts as described for Fig 2, including the additional calculated distribution for the weight-average molar mass, M_w_. It can be seen that the extracts are polydisperse samples with high M fractions. For oat (Fig 3), M_w_ ranges between 10^5^–10^8^ g/mol, the r_rms_ ranges between 50 and 120 nm. For barley (Fig 4) the values are in the same range for M_w_ but have a rather wider range of r_rms_ (20 to 140 nm). Mean values for M_w_ and r_rms_ for both extracts as well as their respective mass recoveries from the AF4 separation channel are summarized in Table 1. These results show that oat β-glucan is larger than the barley β-glucan which is in agreement with earlier findings i.e. by Beer, Wood, & Weisz [43] and Ahmad, Anjum, Zahoor, Nawaz, & Dilshad [44]. The differential M distribution of the extracts, shown in Fig 5, also indicates barley β-glucan as smaller compared to oat β-glucan and that both extracts contain high M species.

**Fig 6:**
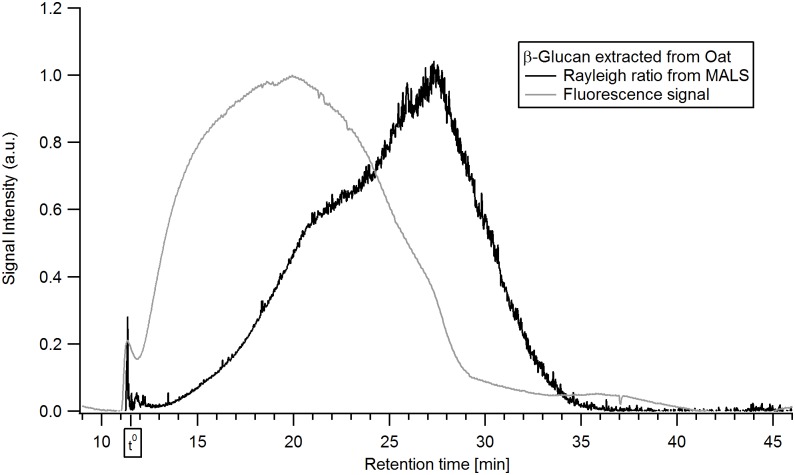
Fluorescence-fractogram of the β-glucan extract from oat. The colors denote: black—Rayleigh ratio from MALS (a.u.), grey—fluorescence signal of in-line Calcofluor-labelled β-glucan at λ_em_ = 445 nm (a.u.).

**Fig 7:**
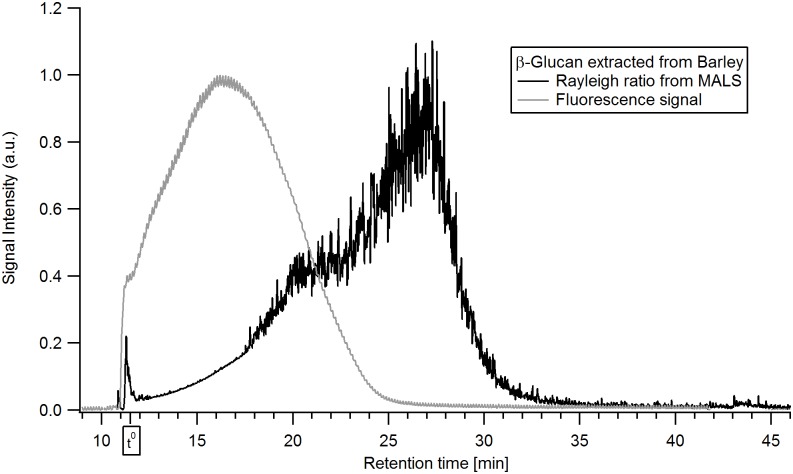
Fluorescence-fractogram of the β-glucan extract from barley. The colors denote: black—Rayleigh ratio from MALS (a.u.), grey—fluorescence signal of in-line Calcofluor-labelled β-glucan at λ_em_ = 445 nm (a.u.).

Utilizing AF4 in combination with FL detection, β-glucan could be successfully labelled and made visible in the fractograms. The results of combining the AF4-MALS with selective FL labelling of the β-glucan can be seen in Fig 6 for oat and Fig 7 for barley. The signals were normalized against the peak maxima. A short delay in the elution times compared to the AF4-MALS-dRI was observed owing to different tubing configuration to allow the in-line labelling procedure. The β-glucan in the extracts have been selectively labelled with Calcofluor and detected at a wavelength of λ_em_ = 445 nm.

The results show the presence of β-glucan over the entire size distribution for oat β-glucan (Fig 6) while the late eluting analytes (retention time >25 min) in the barley β-glucan (Fig 7) sample appear to consist of low amounts of β-glucan or no β-glucan at all. The interpretation of Calcofluor labelled β-glucan data has some limitations as low M β-glucan (M_w_ approx. 4·10^4^ g/ mol) tends to yield a lower signal intensity which for the low M analytes is also dependent on the ionic strength [45]. This might lead to underestimation of the content of low M β-glucan in the sample. For the late eluting analytes the low or non-existent FL-signal also coincides with a low dRI-signal (i.e. low concentration of analytes) in Figs 3 and 4 making it unclear whether the β-glucan content is lower or it is just a consequence of the very low analyte concentration. HPAEC-PAD showed xylose and arabinose, which are products of arabinoxylan hydrolysis, in small amounts (Table 1) when compared to data available in literature [46]. It is possible that the late eluting analytes (>25 min) in the barley extract are traces of very large size arabinoxylan species present at very low concentration and with no FL-signal but with distinct MALS-signal (Figs 3 and 7). These are also apparent in xylanase 11 digests of barley extract run on PACE gels (Fig 1). However, it is not possible to be conclusive that this is the case based on the results obtained.

Since a presence of protein in the β-glucan system would play an important role in formation of aggregates and could contribute to the claimed health benefits of β-glucan both extracts were analysed for protein content using two different approaches: AF4-MALS-FL (with pre-injection fluorescent labelling) and total N content determination through elemental analysis. AF4-MALS-FL did not show the presence of any proteinaceous moieties in either extracts (data not shown). Similarly, the elemental analysis showed no detectable amounts of nitrogen/protein (detection limit approx. 100 PPM). Although using an extraction procedure with mild conditions we found no evidence supporting the hypothesis of proteinaceous matter bound or creating a complex or aggregate with oat or barley β-glucan.

## Conclusion

Mild extraction conditions were used to isolate β-glucan from oat and barley flour without modifying their original structure. The method gave β-glucan of good yield and purity. The main impurities are starch and possible starch degradation products and trace amounts of arabinose, xylose, mannose, galactose and fucose. The extracts contained β-glucan of high M_w_ (10^5^–10^8^ g/ mol) and large size (r_rms_ from 20 to 120 nm for oat, up to 140 nm for barley). Furthermore, the extracts were analysed for possible proteinaceous moieties in the β-glucan. There were no covalently bound proteins found in the β-glucan extracts produced by the method described and therefore this study does not strengthen the hypothesis of covalently bound proteins to β-glucan. Hence, the suggested importance of proteinaceous moieties for the functionality of β-glucan and their beneficial health effects cannot be supported.

## Abbreviations

AF4: asymmetric flow field-flow fractionation
ANTS: 8-aminonaphtalene-1,3,6-trisulphonic acid
BSA: bovine serum albumin
DP: degree of polymerization
dRI: differential refractive index
EDAC: 1-ethyl-3-(3-dimethylaminopropyl)carbodiimide
HPAEC-PAD: high-performance anion-exchange chromatography equipped with pulse-amperometric detector
MALS: multiangle light scattering
M: molar mass
M_w_: weight-average molar mass
N: nitrogen
PACE: polysaccharide analysis by carbohydrate gel electrophoresis
r_rms_: root-mean-square radius
RT: room temperature, 20°C
t^0^: void time
TEMED: N,N,N′,N′—tetramethylethylenediamine
TFA: tetrafluoro amide
(Xyl)_1–6_: xylo-oligosaccharides, DP 1–6

## References

[1] U.S. Food and Drug Administration. Food Labeling: Health Claims; Soluble Fiber from Whole Oats and Risk of Coronary Heart Disease. Federal Register. 1992;62: 15343–15344.

[2] U.S. Food and Drug Administration. Food Labeling: Health Claims; Soluble Fiber from Certain Foods and Risk of Coronary Heart Disease. Federal Register. 2008;73: 47828–47829. 18956498

[3] European Food Safety Authority. Scientific Opinion on the Substantiation of Health Claims related to Beta-Glucans and Maintenance of Normal Blood Cholesterol Concentrations (ID 754, 755, 757, 801, 1465, 2934) and Maintenance or Achievement of a Normal Body Weight (ID 820, 823) pursuant to Article 13(1) of Regulation (EC) No 1924/2006. EFSA Journal. 2009;7.

[4] European Food Safety Authority. Scientific Opinion on the Substantiation of a Health Claim related to Oat Beta-Glucan and Lowering Blood Cholesterol and Reduced Risk of (Coronary) Heart Disease pursuant to Article 14 of Regulation (EC) No 1924/2006. EFSA Journal. 2010;8.

[5] Wolever TMS, Tosh SM, Gibbs AL, Brand-Miller J, Duncan AM, Hart V, et al Physicochemical properties of oat β-glucan influence its ability to reduce serum LDL cholesterol in humans: a randomized clinical trial. Am J Clin Nutr. 2010;92: 723–732. 10.3945/ajcn.2010.29174 20660224

[6] Gómez C, Navarro A, Gamier C, Horta A, Carbonell JV. Physical and structural properties of barley (1→3),(1→4)-β-D-glucan Part III. Formation of aggregates analysed through its viscoelastic and flow behavior. Carbohyd Polym. 1997;34 (3): 141–148.

[7] Wood PJ. Relationships between solution properties of cereal b-glucans and physiological effects—a review. Trends Food Sci Tech. 2004;15: 313–320.

[8] Cui SW, Wang Q. Cell wall polysaccharides in cereals: chemical structures and functional properties. Struct Chem. 2009;20: 291–297.

[9] Håkansson A, Ulmius M, Nilsson L. Asymmetrical flow field-flow fractionation enables the characterization of molecular and supramolecular properties of cereal beta-glucan dispersions. Carbohyd Polym. 2012;87: 518–523. 10.1016/j.carbpol.2011.08.014 34662997

[10] Lazaridou A, Biliaderis CG. Molecular aspects of cereal beta-glucan functionality: Physical properties, technological applications and physiological effects. J Cereal Sci. 2007;46 (2): 101–118.

[11] Forrest IS, Wainwright T. Mode of binding of beta-glucans and pentosans in barley endosperm cell-walls. J I Brewing. 1977;83: 279–286.

[12] Webster FH, Woods PJ. Oats: Chemistry and Technology. 2nd ed AACC International Inc., Minnesota, USA; 2011.

[13] Autio K, Myllymaki O, Suortti T, Saastamoinen M, Poutanen K. Physical properties of (1→3),(1→4)-beta-D-glucan preparates isolated from finnish oat varieties. Food Hydrocolloid. 1992;5: 513–522.

[14] Vårum KM, Smidsrød O. Partial chemicial and physical characterisation of (1→3),(1→4)-β-D-glucans from oat (Avena sativa L.) aleurone. Carbohyd Polym. 1988;9: 103–117.

[15] Acker L, Diemair W, Samhammer E. The lichenin of oats. 1. Properties, preparation and composition of the muciparous polysaccharides. Z Lebensm Unters For. 1955;100: 180–188.

[16] Johansen HN, Wood PJ, Knudsen KEB. Molecular weight changes in the (1→3),(1→4)-beta-D-glucan of oat incurred by the digestive processes in the upper gastrointestinal tract of pigs. J Agr Food Chem. 1993;41: 2347–2352.

[17] Zhou M, Robards K, Glennie-Holmes M, Helliwell S. Effects of enzyme treatment and processing on pasting and thermal properties of oats. J Sci Food Agr. 2000;80: 1486–1494.

[18] Dawkins NL, Nnanna IA. Oat gum and β-glucan extraction from oat bran and rolled oats: Temperature and pH effects. J Food Sci. 1993;58: 562–566.

[19] Wood PJ, Paton D, Siddiqui IR. Determination of β-glucan in oats and barley. Cereal Chem. 1977;54 (3): 524–533.

[20] Ahmad A, Anjum FM, Zahoor T, Nawaz H, Ahmed Z. Extraction and characterization of beta-D-glucan from oat for industrial utilization. Int J Biol Macromol. 2010;46 (3): 304–309. 10.1016/j.ijbiomac.2010.01.002 20083136

[21] Nilsson L. Separation and characterization of food macromolecules using field-flow fractionation: A review. Food Hydrocolloid. 2013;30: 1–11.

[22] Giddings JC. Field-flow fractionation: Analysis of macromolecular, colloidal, and particulate materials. Science. 1993;260: 1456–1465. 8502990 10.1126/science.8502990

[23] Tügel I, Runyon JR, Gomez Galindo F, Nilsson L. Analysis of polysaccharide and proteinaceous macromolecules in beer using asymmetrical flow field-flow fractionation. J I Brewing. 2015;121: 44–48.

[24] Ulmius M, Adapa S, Önning G, Nilsson L. Gastrointestinal conditions influence the solution behaviour of cereal β-glucans in vitro. Food Chem. 2012;130: 536–540.

[25] Ulmius M, Önning G, Nilsson L. Solution behavior of cereal β-glucan as studied with asymmetrical flow field-flow fractionation. Food Hydrocolloid. 2012;26: 175–180.

[26] Wahlund KG, Giddings JC. Properties of an asymmetrical flow field flow fractionation channel having one permeable wall. Anal Chem. 1987;59 (9): 1332–1339. 3605623 10.1021/ac00136a016

[27] Ajayi AO, Fagade OE. Heat activation and stability of amylases from Bacillus species. Afr J of Biotechnol. 2007;6 (10): 1181–1184.

[28] Kosik O, Bromley JR, Busse-Wicher M, Zhang Z, Dupree P. Studies of enzymatic cleavage of cellulose using polysaccharide analysis by carbohydrate gel electrophoresis (PACE) In: Gilbert HJ editor. Cellulases. Elsevier Academic Press Inc: San Diego, USA; 2012 pp. 51–67. 10.1016/B978-0-12-415931-0.00004-5 22608721

[29] Goubet F, Barton CJ, Mortimer JC, Yu X, Zhang Z, Miles GP, et al Cell wall glucomannan in Arabidopsis is synthesised by CSLA glycosyltransferases, and influences the progression of embryogenesis. Plant J. 2009;60: 527–538. 10.1111/j.1365-313X.2009.03977.x 19619156

[30] Håkansson A, Magnusson E, Bergenståhl B, Nilsson L. Hydrodynamic radius determination with asymmetrical flow field-flow fractionation using decaying cross-flows. Part I. A theoretical approach. J Chromatogr A. 2012;1253: 120–126. 10.1016/j.chroma.2012.07.029 22835686

[31] Alftrén J, Peñarrieta JM, Bergenståhl B, Nilsson L. Comparison of molecular and emulsifying properties of gum arabic and mesquite gum using asymmetrical flow field-flow fractionation. Food Hydrocolloid. 2012;26 (1): 54–62.

[32] Berry GC. Thermodynamic and conformational properties of polystyrene. I. Light-Scattering studies on dilute solutions of linear polystyrenes. J Chem Phys. 1966;44 (12): 4550–4564.

[33] Andersson M, Wittgren B, Wahlund KG. Accuracy in multiangle light scattering measurements for molar mass and radius estimations. Model calculations and experiments. Anal Chem. 2003;75 (16): 4279–4291. 14632147 10.1021/ac030128+

[34] Li W, Cui SW, Wang Q, Yada RY. Studies of aggregation behaviours of cereal b-glucans in dilute aqueous solutions by light scattering: Part I. Structure effects. Food Hydrocolloid. 2011;25: 189–195.

[35] Immerstrand T, Bergenståhl B, Trägårdh C, Nyman M, Cui S, Öste R. Extraction of β-Glucan from Oat Bran in Laboratory Scale. Cereal Chem. 2009;86 (6): 601–608.

[36] Papageorgiou M, Lakhdara N, Lazaridou A, Biliaderis CG, Izydorczyk MS. Water extractable (1→3,1→4)-β-D-glucans from barley and oats: An intervarietal study on their structural features and rheological behavior. J Cereal Sci. 2005;42: 213–224.

[37] Robyt JF. Enzymes and their action on starch In: Bemiller JN, Whistler RL, editors. Starch: Chemistry and Technology. Academic Press: Elsevier, London, UK; 2009 pp. 238–292

[38] Alves LA, Almeida e Silva JB, Giulietti M. Solubility of D-Glucose in Water and Ethanol/ Water Mixtures. J Chem Eng Data. 2007;52: 2166–2170.

[39] Gong X, Wang C, Zhang L, Qu H. Solubility of Xylose, Mannose, Maltose Monohydrate, and Trehalose Dihydrate in Ethanol-Water Solutions. J Chem Eng Data. 2012;57: 3264–3269.

[40] Caldwell KD, Nguyen TT, Myers MN, Giddings JC. Observations on anomalous retention in steric field-flow fractionation. Separ Sci Technol. 1979;14 (10): 935–946.

[41] Andersson M, Wittgren B, Wahlund KG. Ultrahigh molar mass component detected in ethylhydroxyethyl cellulose by asymmetrical flow field-flow fractionation coupled to multiangle light scattering. Anal Chem. 2001;73: 4852–4861. 11681461 10.1021/ac0104734

[42] Perez-Rea D, Bergenståhl B, Nilsson L. Development and evaluation of methods for starch dissolution using asymmetrical flow field-flow fractionation. Part I: amylopectin. Anal Bioanal Chem. 2015;407: 4315–4326. 10.1007/s00216-015-8611-8 25925852

[43] Beer MU, Wood PJ, Weisz J. Molecular weight distribution and (1→3)(1→4)-β-D-glucan content of consecutive extracts of various oat and barley cultivars. Cereal Chem. 1997;74: 476–480.

[44] Ahmad A, Anjum FM, Zahoor T, Nawaz H, Dilshad SMR. Beta Glucan: A Valuable Functional Ingredient in Foods. Crit Rev Food Sci. 2012;52: 201–212. 10.1080/10408398.2010.499806 22214441

[45] Kim S, Inglett GE. Molecular weight and ionic strength dependence of fluorescence intensity of the Calcofluor/ β-glucan complex in flow-injection analysis. J Food Compos Anal. 2006;19: 466–472.

[46] Izydorczyk MS, Dexter JE. Barley β-glucans and arabinoxylans: Molecular structure, physicochemical properties, and uses in food products—a Review. Food Res Int. 2008;41: 850–868.

